# The Food (Promotion and Placement) regulations are beginning to shift the onus for healthier choices from individuals to businesses: in-depth perspectives from health experts

**DOI:** 10.1186/s12916-025-04484-2

**Published:** 2025-12-17

**Authors:** Preeti Dhuria, Sarah Muir, Amelia Bird, Wendy Lawrence, Emma Roe, Janis Baird, Christina Vogel

**Affiliations:** 1 Medical Research Council Lifecourse Epidemiology Centre, University of Southampton, Southampton General Hospital, Tremona Road, Southampton, SO16 6YD UK; 2 Primary Care, Population Science and Medical Education, Faulty of Medicine, University of Southampton, Highfield Campus, Southampton, SO17 1BJ UK; 3 School of Geography and Environmental Science, University of Southampton, Highfield Campus, Southampton, SO17 1BJ UK; 4 National Institute for Health Research Southampton Biomedical Research Centre, University of Southampton, and University Hospital Southampton NHS Foundation Trust, Tremona Road, Southampton, SO16 6YD UK; 5 NIHR Applied Research Collaboration Wessex, Southampton Science Park, Innovation Centre, 2 Venture Road, Chilworth, Southampton, SO16 7NP UK; 6 Centre for Food Policy, City St George’s, University of London, Northampton Square, London, EC1V 0HB UK

**Keywords:** Less-healthy foods and drink, Health experts, Qualitative analysis, Food environment, Public health policy, Promotion and placement, HFSS

## Abstract

**Background:**

Retail food environments in the UK use intense marketing strategies to promote the purchase and consumption of less-healthy foods that are associated with ill-health. To help address this issue, the Food (Promotion and Placement) regulations were introduced in England from October 2022, banning the placement of foods high in fat, salt, or sugar (HFSS) at checkouts, aisle-ends, and entrances in qualifying retail settings. Ahead of their introduction, this study examined health experts’ (i) perspectives on the likely effectiveness of these regulations and (ii) recommendations to enhance their impact.

**Methods:**

This cross-sectional qualitative study aimed to recruit health experts to partake in focus groups/semi-structured interviews via MS Teams. Data were collected, coded, and analysed by three researchers with input from senior colleagues, using Braun and Clarke’s reflexive thematic analysis method.

**Results:**

Data were collected between October 2021 and March 2022 from 28 health experts, including public health and food policy academics (*n* = 9) and experts from civil society organisations (*n* = 19). Health experts perceived regulations as a major policy innovation which recognised businesses’ role in driving poor dietary choices that contribute to obesity. They also raised concerns about the outdated nutrient profiling model, limited regulatory scope, and weak enforcement. They were apprehensive about the potential for disproportionate impacts on smaller businesses and certain consumer groups. To enhance the impact of the regulations, they recommended funding independent and diverse evaluations, mandating the reporting of business sales data, and strengthening enforcement efforts. To improve the regulations’ effectiveness, they also suggested establishing mechanisms to refine regulatory guidance and introducing complementary policies within the food system.

**Conclusions:**

Health experts believed that the regulations represent a significant step to curb the promotion of unhealthy foods in retail environments but will be insufficient on their own to improve population diet. To maximise their impact, a systems approach is essential, addressing shortcomings of the regulations, supporting smaller retailers in adopting health initiatives, and implementing thorough monitoring and evaluation. The regulations must form part of a comprehensive set of policies across various sectors, including manufacturing and retail, to accelerate food system transformation and address the dietary drivers of ill-health.

**Supplementary Information:**

The online version contains supplementary material available at 10.1186/s12916-025-04484-2.

## Background

Obesogenic environments are a significant driver of poor dietary patterns in the UK [1]. The majority of adults and children in the UK exceed the recommended levels of sugar, salt, and saturated fat intake [2]. Additionally, the diets of significant proportions of adults and children (75% and 90% respectively) fall short of national guidelines for fruit, vegetable, and fibre consumption [3]. These dietary imbalances contribute to the high prevalence of obesity, which in turn leads to a number of chronic health issues including diabetes, cardiovascular disease, and cancer [4]. Despite policy advancements over the last three decades, obesity remains widespread, affecting around 67% of men, 60% of women, 22% of children (10–11 years), and 19% of adolescent (11–15 years), underscoring the growing public health concern in the UK [5, 6]. Moreover, obesity and its associated health outcomes are socioeconomically patterned, with those most disadvantaged experiencing the greater burden of illness [7–9]. The escalating cost of living has further compounded these inequalities, hindering the ability for families living on low incomes to access nutritious food and perpetuating the cycle of financial insecurity, poor diet, and ill-health [10].

Shifting dietary patterns to address obesity is complex, as many interrelated factors across the whole food system play a role [11]. The socio-ecological model (SEM) highlights that dietary behaviours are shaped not just by individual choices, but also by broader interpersonal, organisational, community, and policy-level influences [12]. Despite this, government efforts have frequently relied on downstream strategies such as nutrition education and labelling that place responsibility on individuals, while overlooking upstream determinants of choice [13]. For example, the UK’s mandatory back-of-pack labelling under EU regulation No. 1169/2011 implemented from Dec 2016 and voluntary front-of-pack traffic light system officially recommended by Health Ministers in 2013 to support individuals make informed choices based on the information provided on pre-packaged foods [14, 15]. However, research shows that nutritional labels often have limited impact and may exacerbate health inequalities, particularly among families facing time and financial constraints, or who have English as a second language [16, 17]. By contrast, upstream interventions, which alter the food environment to make healthier options the default, align more closely with the SEM’s emphasis on structural influences. One such example of upstream policy intervention is the UK’s mandatory Soft Drinks Industry Levy (SDIL) which has prompted the food industry to reformulate products and reduce portion sizes resulting in lower sugar consumption at the population level [18, 19]. Combining downstream interventions with upstream strategies that address structural factors is likely to be most effective at curbing obesity rates because the level of individual agency and resources required to make healthful choices is reduced [20].

Food and beverages high in fat, salt, or sugar (HFSS) have been identified as the biggest contributors to high calorie intakes [21]. Moreover, these foods are highly profitable for businesses due to their relatively low production costs, long shelf life, and strong consumer appeal, driven by ingredients like sugar, salt, and fat [22]. Retail stores are strategically crafted to encourage impulse purchases, with in-store promotions dominated by HFSS products [23–25], and these less-healthy items placed at checkouts and other prominent locations in stores [26, 27]. Consumers’ food shopping decisions are predominantly made in these settings, where the availability, price, promotion, and placement of food options significantly influence choices [28, 29]. Despite individuals and families frequently intending to select healthier food options, the design of retail outlets prompts alternative or additional choices [9, 30]. Evidence in the UK demonstrates that a significant volume of HFSS products promoted in prominent locations in retail settings appeals to both children and adults, prompting impulse purchases and increased consumption [31, 32]. Implementing healthier in-store marketing and placement strategies therefore has strong potential to promote healthier food choices effectively and equitably [25, 27, 33]. The term ‘consumers’ is used in this paper in an inclusive and active way recognising the voice and independence people can have over their food choices.

In addition to layout, the type of store itself influences dietary patterns by shaping what foods are available and accessible. Smaller convenience stores often offer a limited selection of healthy food options, while stocking more processed and high-calorie items [34, 35]. This restricted availability can exacerbate health inequalities, particularly for low-income individuals who depend on these outlets because of transportation or geographic barriers that limit access to larger supermarkets [36, 37]. As a result, retail settings of all types, including supermarkets and convenience stores, are key obesogenic environments and critical targets for policy interventions aimed at improving diets [38, 39].

**Fig. 1:**
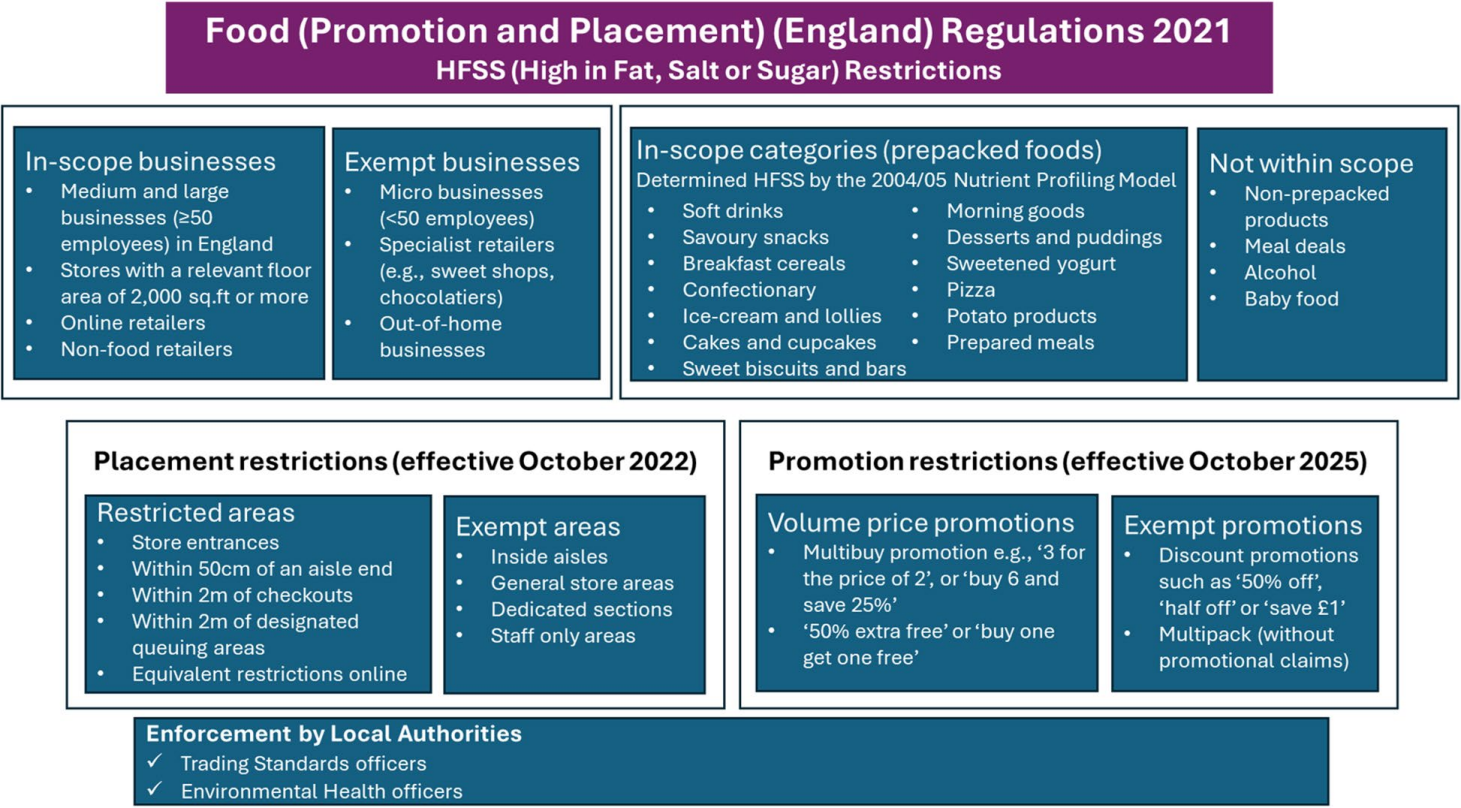
Key provisions of the regulations

In 2022, the UK government implemented the Food (Promotion and Placement) regulations (*hereafter the regulations*) to reduce the promotion and visibility of unhealthy foods to consumers, particularly children [40]. The regulations apply to medium and large retailers (with 2000 square feet relevant floor sales area or more and with 50 or more employees), offering prepacked food for sale in-store and online, including non-food, franchise, and symbol group stores [41]. Under the regulations, prepacked foods that score 4 or more and drinks that score 1 or more on the 2004–2005 Nutrient Profile Model (NPM) are considered unhealthy. Restrictions apply to prepacked foods from 13 categories (namely soft drinks, savoury snacks, breakfast cereals, confectionary, ice cream and lollies, cakes and cupcakes, sweet biscuits and bars, morning goods, desserts and puddings, sweetened yogurt, pizza, potato products, and prepared meals including products in sauce and breaded or battered foods). In-store locations where unhealthy products cannot be placed include checkouts, designated queuing area, aisle-ends, store entrances, and covered external areas [41]. Non-prepacked foods, meal deals, small, independent and specialist stores (selling one type of food product such as chocolatiers), and the out-of-home sector such as cafes/cinemas are exempt. The key provisions of regulations are shown in Fig. 1.

Many individuals and organisations play an important role in advocating public policy changes by generating and sustaining political commitment towards regulatory actions [42, 43]. These stakeholders come from diverse backgrounds such as research, academia, and civil society organisations (CSOs), including non-government organisations (NGOs), charities, and advocacy groups, but hold a shared interest in improving public health [43, 44]. The involvement of these stakeholders is crucial to regulatory action, as demonstrated by the tobacco control movement [45], where convergence of rigorous evidence, along with strategic framing and targeted advocacy, successfully shifted the policy focus to public health priorities [45]. Academics contribute through robust research evidence, academic publications, and formal government consultations, while CSOs raise awareness, mobilise public support, and hold policymakers accountable through accessible reports, media coverage, and lobbying politicians [44]. Collectively referred to as health experts in this study, these stakeholders have a vested interest in protecting public health and keeping governments and commercial companies accountable. The actions of these experts are vital in guiding policymakers towards evidence-based approaches that address the needs of all consumers. While health experts have frequently contributed to government consultations regarding obesity-related strategies and regulations, there is a lack of published evidence comprehensively examining the perspectives of health experts from diverse backgrounds on the introduction of these regulations. This study therefore aimed to address this evidence gap by assessing health experts’ perspectives on the efficacy of the landmark regulations and to ensure more equitable outcomes, across all population groups. The specific research questions for this study were as follows: (1) What do health experts perceive will influence the regulations’ effectiveness? 2) What actions do health experts believe should be put in place to enhance the impact of the regulations?

## Methods

### Study design and setting

This study was cross-sectional and qualitative in design. This design was selected to achieve a thorough understanding of perspectives from a diverse range of health experts shortly after the announcement of the regulations. Focus groups were chosen as the preferred data collection method for this study because they facilitate participant interaction which can evoke more nuanced themes [46]. This dynamic was particularly valuable when engaging with health experts, because it enabled a more comprehensive exploration of complex systemic issues surround poor diet and obesity. Given that health experts, particularly academics, possess specialised knowledge in their respective fields, this approach was effective for capturing dynamic exchanges among experts. However, difficulty aligning participant schedules meant offering flexibility in the data collection approach. Health experts joined either a focus group or an interview according to their availability and preference. The setting for this study was England because this covers the jurisdiction of the regulations. Regarding the research team’s positionality, PD (*first author*) identifies as British Asian, while the rest were of White ethnicity, aged between 25 and 62. Collectively, the team’s expertise spanned nutrition, food policy, public health, psychology, and geography. Ethical approval for this study was obtained from the University of Southampton Faculty of Medicine ethics committee (Ethics ID—65,419.A1). The study adhered to the Declaration of Helsinki, Research Governance Framework Health and Social Care, Data Protection Act 2018, and the Consolidated Criteria for Qualitative Research (COREQ) recommendations [47].

### Study population and recruitment

A purposive sampling approach was taken to obtain insights from health experts with interest and expertise in obesity and/or public health policy in the UK. Health experts were identified through prior professional contacts across academia and CSOs as well as from the list of contributors to the implementation consultation (these data were obtained through a freedom of information request made by the research team to the Department of Health and Social Care (DHSC)). Snowball sampling was also employed, where health experts linked the researchers with individuals within their networks who may be interested in taking part. The purposive sampling aimed to cover a broad spectrum of health experts, encompassing different job roles, relevant expertise in public health and health equity and who worked across a range of CSO and academic organisations across England. The target sample size aimed to include 25–30 health experts that would cover the diversity of jobs roles and backgrounds.

An initial email invitation, along with a study information sheet and a question guide, was sent to 60 potential health experts to gauge interest and offer an opportunity for inquiries. Following the initial email invitation, attempts were made to establish contact through both email and, when feasible, phone communication to optimise participation. Individuals who did not respond after two follow-ups were documented as having declined participation. All health experts completed a consent form and short questionnaire (via email or verbally to provide information on their organisation, job title, role, and experience) before the online interview/focus group commenced. They could withdraw from the study at any time before the data were analysed.

### Data collection

Participant recruitment and data collection occurred simultaneously over 6 months (20 October 2021–29 March 2022), after the draft legislation was released in July 2021 but before the detailed implementation guidance in April 2022. The question guide, developed by PD and reviewed by the co-authors, was designed to explore health experts’ perspectives on the regulations. The questions were informed by findings from a qualitative systematic review of previous policies considered or implemented in retail settings to improve diet [48] and the DHSC’s consultation responses to these regulations, as well as research team’s expertise in retail food environment research. Pilot testing of the question guide was not conducted because of time pressures of the pending regulations and because the health experts invited shared similar professional backgrounds with the researchers, holding a common goal of improving population health. It was therefore anticipated that the terminology used in the question guide would be readily understood by the interviewees. A semi-structured approach was used to obtain insights into factors influencing effectiveness of the regulations, monitoring and evaluation of the regulations’ health impact, and future policy refinements (Additional file 1). The semi-structured nature of the question guide allowed the researchers to build on points raised by health experts and adapt their questions using prompts and probes to seek additional detail in both interviews and focus groups [49]. PD and SM conducted and recorded the interviews and focus groups using MS Teams video conferencing software. The interviews ranged between 18 and 53 min and focus groups (3–4 health experts) 41–57 min.

### Data analysis

Recordings were transcribed verbatim, anonymised, and then imported into NVIVO version 14 for coding management. Data were analysed using Braun and Clarke’s reflexive thematic analysis, taking an inductive approach, and guided by the research questions [50]. PD led the analysis by reading all the transcripts to build familiarity with the dataset and inductively coded phrases and sentences across interview and focus group transcripts to ensure balanced representation. The codes were reviewed to assess the relationship with other codes and grouped into clusters. Recurring patterns within the clusters were grouped together to develop six initial themes: (i) These regulations are symbolic but there are regulatory concerns; (ii) Enforcement will be incredibly challenging; (iii) The regulations rely on businesses to adhere to the spirit of legislation; (iv) Consumer acceptability of the regulations may vary; (v) Building evidence for the regulations will reinforce its effectiveness; and (vi) A range of policies is required to enhance regulations’ impact. These initial themes were discussed with WL and CV to ascertain their consistency with the codes and dataset. PD then refined the themes and subthemes to develop an initial coding frame. Another researcher (AB) used the initial coding frame to double code six transcripts (three interviews and three focus groups) for quality assurance and to avoid any dominance of a single researcher’s perspective. PD and AB met twice to discuss the development of codes and themes and agree on any differences in coding. Overall, there was agreement on the coding frame, but some subthemes were altered slightly, and additional data excerpts were extracted following discussions. The themes and subthemes were refined further in discussion with all co-authors to capture the independent narratives for each theme. One participant, a public health researcher based at a different institution, was pragmatically selected to provide an external perspective and to sense-check the findings. This individual was chosen based on their relevant expertise in nutrition policy and health equity, as well as their availability and willingness to engage in the process of confirming the findings. A summary of the results, along with a figure illustrating key themes and subthemes, was shared with the individual, prior to a Teams meeting to discuss the interpretations of findings. During the discussion, the individual elaborated on some of the subthemes. For instance, the subtheme ‘*Ensure funding for diverse and independent evaluations*’ was expanded to emphasise the challenge of measuring systems-level impacts of public health interventions in the short term. Therefore, further detail was incorporated into the results and discussion sections, calling for better funding mechanisms to enable long-term evaluation of the regulations, particularly their sustained effects on shopping patterns or retailer compliance.

**Fig. 2:**
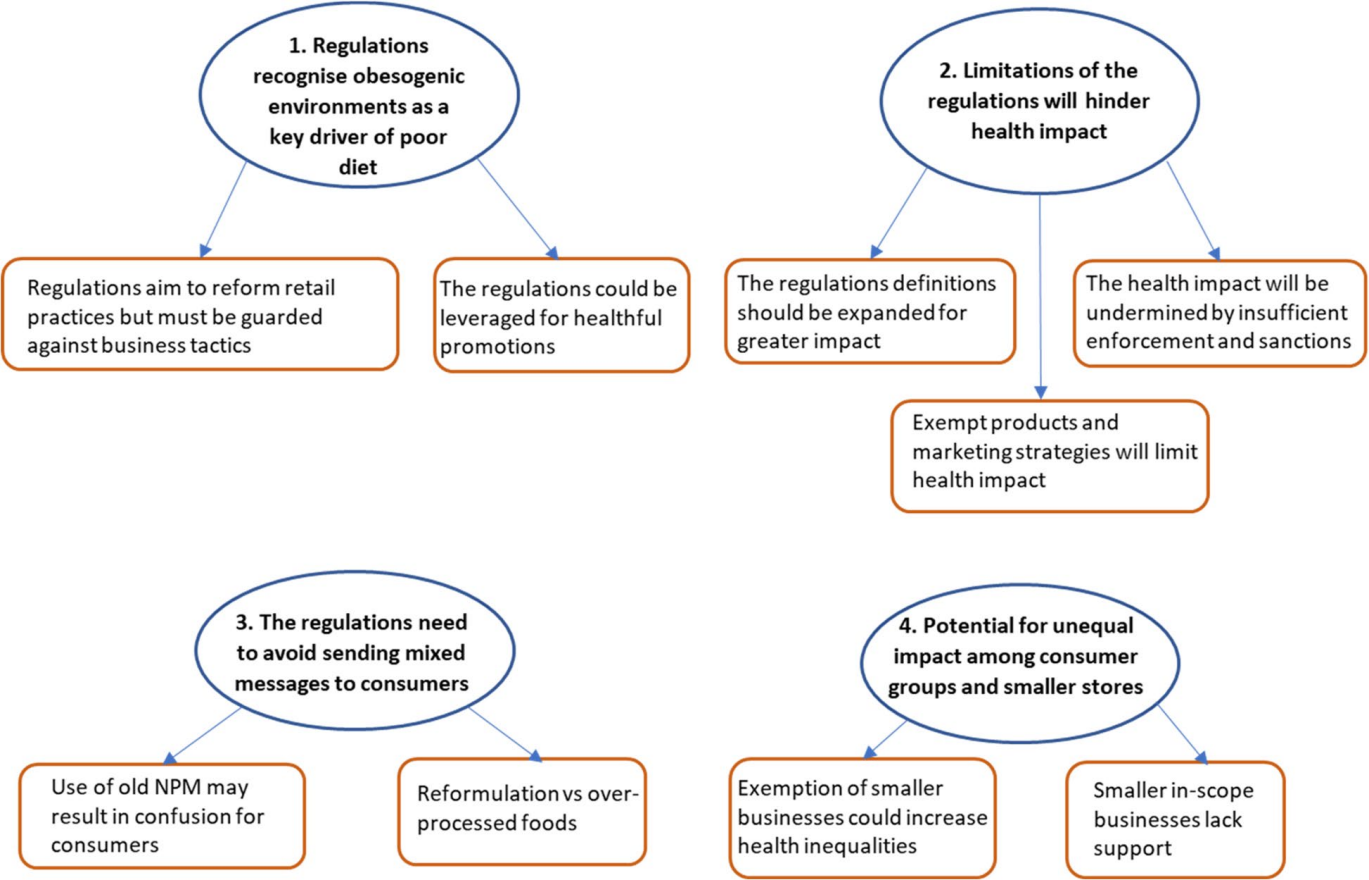
Key themes addressing research question (1) What do health experts perceive will influence the regulations’ effectiveness?

**Fig. 3:**
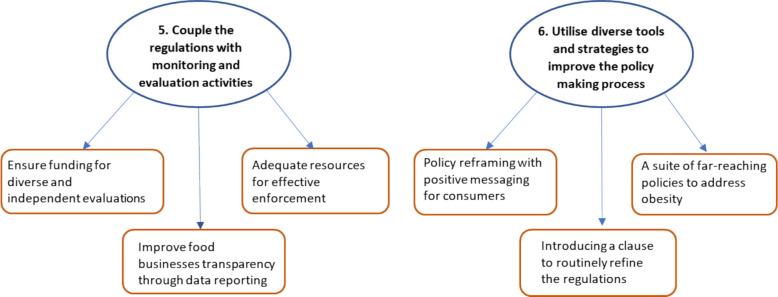
Key themes addressing research question (2) What actions do health experts believe should be put in place to enhance the impact of the regulations?

The findings are organised thematically against the two research questions, with key themes visually depicted in Figs. 2 and 3. Each theme is presented with its subthemes and illustrated with quotes from across interviews and focus groups to reflect the full range of participant voices. Quotes are anonymised using unique identifiers that indicate a unique participant ID, data collection method (interview or focus group), and the participant’s sector (e.g. academic or).

## Results

### Participant characteristics

In total, seven interviews and six focus groups were conducted with 28 health experts whose job roles and type of organisation are shown in Table 1. Of the 28 health experts, nine (32%) were public health, nutrition, and food policy academics, and 19 (68%) were representatives from CSOs such as NGOs, charities, and youth groups engaged in advocacy efforts to combat obesity-related illnesses. A total of 12 (43%) health experts held senior positions in their organisations.

**Table 1:** Health experts’ background and job roles

Participant background	*N* = 28 (%)
Academia (public health, nutrition, and food policy) (*n* = 9)	
Professor/associate professor	4 (14)
Research fellow/associate	4 (14)
Lecturer	1 (3)
CSOs (NGOs, charities, and advocacy groups) (*n* = 19)	
Director/CEO	6 (21)
Senior manager (e.g. head of campaigns)	2 (7)
Manager (e.g. of policy, health)	5 (18)
Professional/other (e.g. nutritionist, campaign coordinator)	6 (21)

### Research question (1) What do health experts perceive will influence the regulations’ effectiveness?

The first four themes (Fig. 2) describing health experts’ perspectives on factors that could influence the effectiveness of the regulations are detailed below with illustrative participant quotes.

#### Theme 1: regulations recognise obesogenic environments as a key driver of poor diet

##### Regulations aim to reform retail practices but must be guarded against business tactics

By introducing the regulations, health experts felt that the government acknowledged its duty of care to population health. They viewed the regulations as an important shift in recognition of a collective, rather than a solely individual, responsibility to making healthier food choices.

“I think the thing we particularly like about it [the regulations] is setting the halo of something needs to be done and it needs to be the responsibility of the food manufacturers and retailers rather than that of individuals.”—12008, Focus group 1 (CSO representative)

“I think it [the regulations] in part helps take the burden off the individual and is announcing that we’ve actually considered collectively what we think good [marketing strategies] needs to look like and what it is that we’re trying to achieve as a society.”—12014, Focus group 4 (Academic)

Health experts emphasised, however, the need to thoroughly understand and pre-empt the tactics industry may adopt which could undermine the regulations. They discussed the importance of learning from past experiences, particularly from tobacco regulations, to have a clear and consistent ask and discourage businesses from trying to subvert the regulations. Health experts also emphasised the need for collaboration among public health professionals to use strategic, well-organised, and effective approaches to protect the legislation.

We need to be really clear on what we want and what we are trying to achieve and move away from the rhetoric that exists from industry that there’s just a cacophony of sound, public health want everything, but they don’t actually know what they need to do. We do. We’ve got clear aspirations, we’ve got clear legislation, we’ve got clear goals on how we will achieve them, and we just need people to listen to us and we need to be able to beat industry at their own game in that respect.—12006, Focus group 2 (CSO representative)

##### The regulations could be leveraged for healthful promotions

Health experts believed that the regulations provide an opportunity for businesses to increase their promotion and visibility of healthier food products. However, they expressed concern that many businesses might not fully embrace the spirit of the regulations or apply them.

When supermarkets put the fruit and vegetables in the prominent entrance areas, the purchases of fruit and vegetables rise and the supermarkets themselves talk about that. Um, it depends how far the companies respond to the letter of the legislation versus the spirit of the legislation.—12017, Focus group 3 (CSO representative)

#### Theme 2: Limitations of the regulations will hinder its health impact

##### The regulations’ definitions should be expanded for greater impact

The 2004–2005 NPM was initially designed to apply to all foods, but health experts expressed frustration that the regulations only restrict certain foods and drink categories, namely those that contribute significant sugar and calories to children’s diets. They noted that many products that contribute to high salt intakes were excluded, such as garlic bread, pies and quiches, cooking sauces, and processed meats narrowing the scope and public health impact of the regulations.

They decided on them based on sort of products that are heavily promoted. But that just really does narrow the scope. A lot of things in the salt reduction categories were not included. From our perspective in terms of blood pressure and a risk factor for heart diseases, we’d really like to see those aspects covered as well as sugary products and things in the calorie reduction programme.—12060, Interview (CSO representative)

Another concern to health experts was the critical gap in the regulations of failing to include prepacked baby foods and snacks. The omission of this category undermines efforts to promote healthy eating from an early age, leaving a vulnerable group exposed to less-healthy food options.

We know that infants and young children, are given commercial baby foods and snacks that aren’t affected by this legislation.—12043, Interview (CSO representative)

##### Exempt products and marketing strategies will limit health impact

Health experts expressed concern that many large retailers were adapting by shifting their promotions to exempt products not covered by the regulations. In particular, non-prepacked baked goods and loose confectionery. They also highlighted that many exempt products, including alcohol, savoury and yogurt-coated nuts, pastry products, and red and processed meats, will still be prominently displayed in supermarkets.

So, you will see much more of Krispy Kremes and individual baked goods like croissants and pain au chocolates and things like that because they will be out of scope. So, they could be at the end of aisles still in supermarkets.—12068, Focus group 6 (CSO representative)

Moreover, multipacks sold as a single outer pack containing six separate packs of HFSS products are only in scope, if their packaging promotes price comparisons (e.g. ‘6 for the price of 4’) or if volume promotions are offered on the multipacks themselves (e.g. ‘buy 2 multipacks, get one free’). Health experts felt that businesses are likely to apply price reductions on HFSS products and increasingly sell HFSS products like crisp packets in multipacks to boost sales.

The large retailers could shift their promotion tactics to promote things that aren’t covered by the guidance, such as temporary price reductions and multi pack offers.—12039, Interview (CSO representative)

Health experts discussed that the regulations primarily restrict HFSS placement in high-visibility areas in stores like the aisle-ends, checkouts, and front of the stores and online equivalents. However, areas such as in-aisle mobile displays and strips which hang down in front of aisle shelves are not addressed by the regulations and will likely continue to feature prominent placement of HFSS products.

We’ve noted a few areas where location-based promotions will not be part of the legislation, so things like baskets in aisle, it’s [the regulations] very focused on end of aisle, and checkouts and things. But in aisle and sort of strips down the side of aisles, there are definitely things that we could see probably still happening.—12035, Interview (CSO representative)

##### The health impact will be undermined by insufficient enforcement and sanctions

Health experts expressed concerns that efforts to enforce the regulations could be insufficient. Recent budget cuts to local authorities [who are responsible for enforcement] have left them overstretched with limited personnel available to enforce numerous competing legislations. Health experts also highlighted that the sanctions and infringements were not a significant deterrent for many large businesses and that businesses’ compliance levels could wane if profits from marketing unhealthy foods outweighed the fines.

It comes down to people and money. We can have these policies in place and have the documents written and the processes there but if we don’t have enough people to be able to do it [enforcement] effectively then it’s not going to work.—12020, Focus group 3 (Academic)

You just wonder whether anything that doesn’t have proper penalties is even a law... I think we just do need some more teeth in these policies.—12060, Interview (CSO representative)

Health experts cautioned against misinterpreting enforcement failures as the regulations being ineffective. They argued that effective enforcement was necessary to enable future policy refinement and action on tackling poor dietary behaviours that contribute to obesity.

I think the risk is that if you implement legislation and then it’s not enforced correctly, you might falsely conclude that it didn’t work when the problem wasn’t the legislation, it was the way it was applied. That would be a sad outcome if we reached the false conclusion about it [effectiveness of regulations] and that was used to inform future policy or lack of future plans.—12026, Interview (Academic)

#### Theme 3: The regulations need to avoid sending mixed messages to consumers

##### Use of old NPM may result in confusion for consumers

Health experts highlighted that the 2004–2005 NPM used to define HFSS products in the regulations is outdated and not aligned with current UK dietary guidelines on free sugars and fibre. In 2016, the UK government updated its dietary guidelines, reducing recommendations for free or added sugars to be less than 5% of total dietary energy and increasing recommendations for fibre to 30 g per day [51]. The NPM was modified to incorporate current dietary recommendations and consulted on publicly in 2018 but never implemented [52]. Health experts expressed concern that using the 2004–2005 NPM allows many high added-sugar products to be classified as non-HFSS, undermining the regulations’ intended public health impact.

The nutrient profile model needs to better reflect new government guidance around the need to increase fibre intake and the need to reduce free sugar intake. The new [draft] model rewards high fibre, but in a greater extent punishes sugar content, particularly free sugar content.—12040, Interview (CSO representative)

Health experts noted relying on the outdated 2004–2005 NPM could enable some businesses to create a misleading ‘health halo’ around certain products and confuse consumers’ perceptions of product healthfulness [53]. They highlighted the need for the regulations to be refined and the draft 2018 NPM to be adopted. This change in the definition of food healthfulness could help prevent businesses from marketing only marginally improved products that still do not meet current dietary recommendations.

Technically, under these rules something like Coco Pops could still be marketed at the end of the aisle if it falls within the nutritional profiling guidelines. You know that’s not an intuitively healthy product to a consumer.—12015, Focus group 2 (CSO representative)

##### Reformulation vs over-processing of foods

Health experts anticipated that the regulations may expedite widespread reformulation of HFSS products in order to meet the new regulatory requirements. They highlighted instances where businesses had previously acted swiftly to reformulate products in response to government regulations, such as the sugary drinks levy [19].

We have seen how they [businesses] are able to pivot towards reformulation of sugary drinks in a record amount of time after we were told it was so impossible. It [the regulations] may actually push them towards reformulation quicker than they anticipated.—12006, Focus group 2 (CSO representative)

Some health experts raised concerns that businesses may promote healthier versions of HFSS products and increase their use of nutrient claims on marginally healthier products. Although a product may be promoted as being lower in sugar, it could still be calorie dense and pose health consequences. They hoped that the regulations would not encourage only new products portfolios but rather refine the regular products to have a healthier nutrient profile.

I do think when you are talking about being able to trumpet lower sugar versions, you do then potentially fall into the health claims piece, where it can be a marketing tool for things that are still, yes, there’s a lot less sugar, but what about the calories? … Because what has replaced that sugar?—12035, Focus group 5 (CSO representative)

#### Theme 4: Potential for unequal impact among consumer groups and smaller stores

##### Exemption of smaller business could increase health inequalities

Health experts emphasised that unhealthy products and marketing strategies may become more prevalent in smaller stores. The exclusion of small stores from the regulations could disproportionately impact certain consumer groups. They advocated for the elimination of exemptions based on business size to enable healthier retail marketing practices to become a societal norm and reduce inequalities in access to healthier options.

Evidence suggests that some households with lower incomes don’t have as great an access to larger supermarkets. If we’re excluding these [smaller stores], well that just seems like a huge glare because it feels like that’s just kind of contributing to inequalities because people who are able to access the larger stores, they’re going to benefit.—12005, Focus group 3 (CSO representative)

Health experts pointed out that many smaller businesses genuinely want to contribute positively but may lack awareness or resources to do so effectively. They suggested that instead of simply exempting small businesses, they should be supported in familiarisation with guidance, transitioning to changes in stores, scoring products, and identifying ways to address loss of sales from HFSS products.

A lot of people [smaller business] actually do want to do good, but either a) may not know enough, be aware, or b) they just won’t have the capacity and the time to do it. And I do think that actually there is a missed program of work here. Rather than just saying we are supporting SMEs (small and medium enterprises) by making them exempt, what is the program for a bringing them in?—12040, Interview (CSO representative)

##### Smaller in-scope businesses lack support

Health experts felt the inclusion of franchisee-operated smaller stores (convenience stores operating under a chain brand like Nisa and Premier) in the regulations was beneficial but were concerned about lack of support they would receive. Unlike large supermarket chains, franchisee stores are unlikely to be supported by their head office and this discrepancy creates an inequality in implementation of the regulations which may have health impacts. Health experts suggested that the government could provide more active support to smaller in-scope stores to ensure they meet regulatory requirements and not compromise the health of disadvantaged communities that the regulations are trying to help.

We were pleased to see franchises being included in that, but that then does open up a little bit of a disparity between smaller sized stores that are part of the franchise.—12017 Focus group 3 (CSO representative)

In franchised business, where there’s maybe not standardised operational procedures. You worry that those in the poorer, more deprived areas would be less compliant for whatever reason whether there was more, smaller stores or I don’t know, but you would be worried about the inequalities.—12003, Focus group 4 (Academic)

### Research question (2) What actions do health experts believe should be put in place to enhance the impact of the regulations?

The remaining two themes (Fig. 3) highlight approaches health experts recommended could enhance the impact of the regulations on population health. The theme descriptions are illustrated with participant quotes.

#### Theme 5: Couple the regulations with monitoring and evaluation activities

##### Ensure funding for diverse and independent evaluations

Health experts stressed the importance of undertaking robust and independent evaluations of the regulations’ effectiveness. According to health experts, evaluations should incorporate a range of metrics to provide a systems understanding of effectiveness and identify any unintended consequences. Health experts recommended evaluations include assessments of (i) compliance in-stores through surveys or using planograms; (ii) changes in purchasing behaviour of in-scope as well as exempt categories to understand possible substitution effects; and (iii) track reformulation activities across HFSS products to monitor shifts in nutrient profiles.

We do need really good, robust evaluation of these [regulations] so that we can continue to make the case for a) seeing that it is having an impact on public health and then b) keeping up the movement towards healthier environments that has been outlined as a government priority.—12035 Interview (CSO representative)

There was recognition that evaluating the regulations’ direct impact on food choices would be difficult due to the multiple influences on food choices, other policies in place, and gradual shifts in population dietary behaviours. Health experts called for improved funding mechanisms that enabled longer-term evaluation to properly measure system impacts and track longer-term marketing activities. They also emphasised the need to consider businesses’ financial performance and differences in their ability to adapt to regulatory changes.

I feel like understanding the success metrics will be broad, making sure that we don’t just evaluate health outcomes as the only positive, but also the potential fact that the shares of certain companies aren’t falling, they’re still making profit, they’re diversifying. Evaluation results should be such that they speak to multiple audiences about what success looks like for this kind of intervention.—12067, Focus group 5 (Academic)

Assessing lived experiences of various groups affected by the regulations was also deemed an essential part of any evaluation to ensure additional policy provisions or refinement could be made where necessary.

You definitely would need qualitative monitoring to see, you know, touch on how this has affected different groups and what they end up with in their food basket at the end of a shop, particularly lower income groups.—12015, Focus group 2 (CSO representative)

##### Improve food businesses’ transparency through data reporting

Participants noted that the announcement of the regulations has prompted businesses to improve their data systems to enable inventory mapping of products and their nutrient profiles. They believed that this situation creates a significant opportunity to push for mandatory, transparent, and public reporting of businesses sales data, as recommended in the National Food Strategy [1]. They outlined that businesses should be required to report sales volume of HFSS foods versus healthy metrics, such as fruits and vegetable sales volumes, rather than percentage change which could be subject to data manipulation.

A good food bill that means that companies have to declare such data, but being able to have all companies, especially large ones above 250 employees, declaring what sales they have on each of these parts [sales of HFSS foods, sales of fruit and vegetables, sales by protein type, sales of major nutrients, total food and drinks sales, food waste] and what sales and promotions [on healthy vs unhealthy foods], is exactly the kind of thing we need to measure that.—12067, Focus group 6 (Academic)

It has to be on volume of sales, not percentage change. It’s very easy to fiddle it if it’s percentage change.—12013, Focus group 6 (Academic)

##### Adequate resources for effective enforcement

Health experts emphasised the necessity of financing, supporting, and empowering enforcement officers to carry out their duties effectively. They felt that providing enforcement officers with a deeper understanding of the public health rationale behind the obesity regulations aiming to restrict unhealthy food marketing and equipping them with appropriate tools, such as a centralised NPM calculator, was essential for effective enforcement. They also asked for appropriate processes to be established to comprehensively document breaches.

There’s the need for additional resources to be put into place in order to be able to effectively support local authorities to conduct the enforcement. Just having enough people there to be able to do it and for all stages of the process to be properly financed rather than just one specific part.—12020, Focus group 3 (Academic)

Participants highlighted the importance of enforcement at both local and national levels. They suggested that local enforcement activities could be strengthened by CSOs and the public actively highlighting instances of businesses’ non-compliance. This collective effort could generate societal pressure on businesses, ensure accountability, and promote adherence to the regulations.

I am sure there is going to be a role for third sector [charity organisations or social enterprises] to do a bit of sort of investigating.—12027, Focus group 5 (CSO representative)

#### Theme 6: Utilise diverse tools and strategies to improve the policy making process

##### Framing the regulations as positive for consumers

Health experts described that the regulations hold the potential to provide benefits for families through reducing pester power (tendency of children to nag their parents for unhealthy foods in response to marketing) and improving health outcomes, particularly for low-income groups. However, to avoid the regulations being perceived as restricting offers and increasing food costs, health experts believed the regulations should be framed with a positive message.

They [disadvantaged groups] are not in a hugely powerful position in terms of purchasing healthy options but they’re still pretty cross about being forced into a corner, so as long as the messages come through that the product and the promotions game is all about making them spend more money and it being wasteful then that could play quite positively.—12013, Interview (Academic)

##### Introducing a clause to routinely refine the regulations

Notably health experts discussed the importance of incorporating provisions in the law that empower ministers to regularly review and address any loopholes or shortcomings of the regulations. This proactive approach will ensure that the regulations remain effective and adapt to the ever-evolving marketing strategies being used.

Yeah, and certainly one of the important things is an enabling clause so that ministers can come back and review the impact after two or four years, and then actively are given the powers to plug loopholes there and then. Without it going right through all the tedious legislative pathway all over again.—12013, Focus group 6 (Academic)

Health experts felt that defining what can be displayed, rather than what cannot, may be important to prevent undue pressure on retailers from manufacturers. They suggested that through policy refinements, prominent locations could be designated for exclusively featuring fresh fruits and vegetables or other healthy foods to enhance promotion of health-promoting foods.

We need to have something specific in there [the regulations] about what can be displayed in those locations as opposed to what can’t. For example, nothing processed to be on those aisle-ends, it has to be fresh perishable goods to imply that it will be fresh fruit and vegetables. And then there’s less chance of there being HFSS foods and even other foods that are ultra-processed.—12006, Focus group 2 (CSO representative)

##### A suite of far-reaching policies to address obesity

Health experts emphasised the need for a suite of far-reaching policies to address population wide obesity. They described the need for a careful policy approach that used a range of tools to create a food system that encourages healthier food choices and improves public health while also allowing businesses to thrive, leading to more effective and balanced outcomes. Many acknowledged that while certain policies, such as the regulations and restrictions on junk food advertising to children are beneficial, broader systemic changes are required for significant change.

I do feel that there is a requirement for a rebalancing in the cost of healthy versus less healthy foods. I mean the way to do that is via taxes and subsidies, whether that’s in agriculture or in manufacturing or in retail.—12025, Interview (Academic)

In addition to a comprehensive approach involving multiple policies, health experts stressed the importance of integrating and aligning various policy initiatives. An integrated policy approach could help ensure policies are not viewed in isolation but as part of a cohesive strategy, creating a unified narrative that strengthens the overall policy impact.

I think the more joining in and direct linking with other policy areas so that there is a joined narrative in the mindset…. If there’s an understanding, with the ask for business mandatory reporting, if that is part of the linking [by integrating and aligning various policy initiatives], just to make sure that this policy [the regulations] is even better seen through… so that it’s not a kind of seen as loads of different policy asks as we go.—12067, Focus group 6 (Academic)

## Discussion

### Principal findings

Health experts who participated in this study viewed the regulations as a significant policy shift, recognising that poor diet is shaped by more than individual choice and signalling a move towards holding food businesses accountable for creating healthier food environments. However, they stressed the need for a continual proactive and comprehensive approach from government to effectively navigate the complex legislative and industry dynamics. Health experts outlined several shortcomings in the regulations (*Themes 2 and 3*) that could limit its effectiveness including (i) the use of an outdated 2004–2005 NPM which could confuse consumers about product healthfulness; (ii) limited coverage of food categories, marketing strategies, and retailers potentially reducing overall impact; (iii) insufficient sanctions and enforcement to prevent non-compliance; and (iv) inadequate support for franchisee and smaller in-scope businesses which could exacerbate existing health inequalities.

Mandatory nutrition reporting, as set out in the National Food Strategy, and a thorough whole-system evaluation of the regulations were deemed essential elements to effectively monitor businesses responses to the regulations. Health experts recommended refining the regulations to close potential loopholes and developing an integrated, long-term, joined-up suite of policies to effectively improve population diet.

### Comparison with previous literature

The results from this study are similar to those from previous qualitative work with academics and charities, which show overwhelming support for restrictions on the marketing of HFSS products. Health experts argue that such bold regulations are necessary to effectively improve purchasing behaviour and reduce pester power for unhealthy foods [54, 55]. However, because profit remains the primary driver for food businesses, accountability through regulatory measures and established frameworks is essential to ensure that profitable practices also incorporate health considerations. Global initiatives such as INFORMAS’ BIA-Obesity (Business Impact Assessment on Obesity and Population-level Nutrition) tool benchmarks food businesses’ policies and commitments on nutrition and obesity prevention [56]. This tool evaluates companies across six key domains including corporate strategy, product formulation, nutrition labelling, product and brand promotion, product accessibility, and relationships with other organisations. Evidence from Australia indicates that benchmarking through BIA-Obesity not only provides transparency but can also encourage both companies and policymakers to prioritise nutrition commitments [57]. In New Zealand, application of the tool revealed that while some companies indicated strategic intentions through corporate nutrition strategies, actual commitments to improving product accessibility and responsible promotions was largely absent [58]. This finding highlights the need for stronger, enforceable commitments across the sector and the importance of regulatory approaches. Pairing these regulations with robust accountability mechanisms could encourage compliance and help realign profitability with broader public health goals.

The results from this study suggest that the regulations could motivate food businesses to improve their product data systems (*detailed under Theme 5*), presenting an opportunity to introduce mandatory reporting of product sales as recommended by National Food Strategy [59]. Implementing mandatory reporting and setting healthiness targets for large retailers could be an equitable approach that could make healthier choices more accessible. The approach has been estimated to reduce obesity rates by 20–25% in the UK [60]. The UK government recently announced they will require large food companies to report on the healthiness of their products, alongside setting future mandatory targets [61]. This announcement marks a world-first initiative to improve transparency and accountability. Our findings are consistent with recommendations from the Food Foundation, which emphasises that the reporting should focus on absolute sales volumes of HFSS and healthier foods rather, than percentage changes, to prevent data manipulation and enable meaningful monitoring [62]. Mandatory reporting and health targets hold strong potential to shift the UK food environment, but they must be transparent, properly enforced, and a part of wider strategy. Sufficient resources for strong monitoring and enforcement are also essential to discourage and detect gaming strategies like shrinking portion sizes or substituting one unhealthy ingredient for another instead of genuinely improving nutritional quality. Safeguards such as appropriate pricing, incentives for reformulations, and support for healthier products in value ranges could improve the accessibility and affordability of reformulated products.

The health experts in our study highlighted a notable gap in the implementation of regulations, specifically the capacity to enforce them effectively. Previous research conducted in 2011–2012 with experts in nutrition, public advocacy, and policymaking examined their views on the introduction of taxation policies on sugar-sweetened beverages and unhealthy foods in Israel. The findings similarly indicated that difficulties in enforcement were perceived as a key barrier to effective policy implementation [63]. Additionally, a 2021 narrative review on regulatory governance dynamics identified key shortcomings in the enforcement of various food policies, including limited enforcement resources, poor mechanisms for managing complaints, and insufficient use of sanctions like fines, which result in ineffective adoption of a policy [42]. Our findings suggest that weak enforcement of the regulations could result in lower compliance and create a misleading perception of the regulations’ ineffectiveness, which may negatively impact the adoption of future obesity policies. A key takeaway from both previous research and our study is the importance of proactively hiring and training personnel before implementation of new policies, to ensure effective and equitable enforcement [64]. Ensuring local authorities have sufficient funding to enable monitoring of compliance with the regulations and effective management of breaches is also consistent with existing research evidence, which underscores the importance of skills and sufficient resources to sustain long-term enforcement efforts [65, 66]. Previous evidence and findings from our study also suggest leveraging the voice of CSOs to highlight business adherence or non-compliance could supplement enforcement efforts [65].

Health experts in this study expressed concern that the regulations may exacerbate inequalities among populations who rely on smaller stores for their groceries and are more vulnerable to the health risks that the regulations aim to mitigate. Smaller stores frequently offer fewer healthy and fresh food options and may have higher prices compared to supermarkets [35, 67], yet smaller franchisee businesses that are in-scope of the regulations may lack the financial capital, space, and staffing capacity to implement store layout changes as described in our sister manuscript that explored business responses [68]. Moreover, they may not be trade body members nor have local authority support through primary authority arrangements leaving them at risk of non-compliance and competitive disadvantage [68]. A study by Winkler et al. revealed that beverage companies employ standardised marketing agreements controlling product placement, pricing, and promotional access across food retailers irrespective of size, but systematically offer much more favourable terms (e.g. financial incentives, better servicing) to large chain retailers, while small, independent stores endure non-negotiable, costlier, and lower-service terms [69]. Small UK retailers may similarly face disproportionate constraints compared to major chains when negotiating HFSS product agreements making compliance with HFSS restrictions more costly or operationally difficult thus potentially reinforcing market inequities under the new regulations. Our findings emphasise the need for a comprehensive work program to identify strategies that support small stores in meeting regulatory requirements. Such strategies may include introducing alternative policies that promote healthier retailing, with targeted support to offset power imbalances and ensure equity in regulatory impact while safeguarding small-store viability and health outcomes.

Furthermore, policy refinements should consider the integration of the regulations with other complementary food policies aimed at promoting healthy and sustainable diets [65]. Evidence shows that complementary policies reinforce each other and address multiple drivers of unhealthy eating in various settings. For instance, multiple policy actions in Chile, such as warning labels, advertising restrictions, and banning unhealthy foods in schools, led to a 23.7% decrease in the purchase of sugar-sweetened beverages and a significant reduction in the marketing of unhealthy foods to children [70]. Similarly, in Mexico, the sugar-sweetened beverage tax resulted in a 7.6% decline in the purchase of these beverages. Its impact was further enhanced by accompanying public awareness campaigns about the health risks of sugary drinks [71]. Alongside retail marketing restrictions, opportunities should also be identified to work with supply chain stakeholders for increasing the variety, affordability, and promotions on healthier options [72]. Moreover, each retail policy should not be treated as an isolated rule but as a part of an adaptive system. Coordinating different retail policies such as these regulations, labelling requirements, reformulation targets, advertising restrictions, potential taxation on HFSS foods, and mandatory business sales reporting could help to create a coherent ‘food‑retail ecosystem’ that provides businesses with a consistent regulatory environment and strengthens efforts to promote healthier diets [68].

### Policy and research implications

Our findings highlight several potential future refinements of the regulations. The first relates to the use of the outdated 2004–2005 NPM to define product healthfulness. The old NPM model was deemed a pragmatic choice for the regulations due to difficulties associated with calculating ‘free sugars’ and fibre content which are often not available on back of pack labels [73]. Health experts support the adoption of the updated 2018 NPM and an extension of the regulations to all HFSS foods in future policy refinements to maximise health benefits of the regulations. The updated NPM has been revised to provide a more accurate and comprehensive assessment of product healthfulness by (i) reducing total energy used as a reference from 2130 to 2000 kcal; (ii) shifting focus from total sugars to free sugars; (iii) recommending no more than 5% of food energy from free sugars; and (iv) increasing recommended intake of fibre from 24 to 30 g per day [51]. Manufacturers should be required to declare free sugar and fibre content on the back of pack label to enable NPM assessment. Alternatively, requiring all HFSS products to be labelled as such could be introduced alongside implementation of the draft 2018 NPM to ease identification of HFSS products for everyone [74]. The current UK NPM and associated labels focus on the nutrient composition of foods, but do not consider the degree of processing or the presence of additives [75]. As a result, businesses reformulating products to pass the current 2004–2005 NPM may inadvertently promote the consumption of products that have undergone increased levels of processing and/or contain artificial sweeteners and additives [76]. Therefore, ongoing refinement of NPMs is essential to incorporate the latest scientific evidence on nutrition, health, and the effects of food processing.

A key finding from this study and recent regulatory interventions highlights the importance of comprehensive, independent evaluations of the regulations’ impacts. For instance, robust evaluations of Chile’s front-of-pack labelling and Mexico’s sugary drink tax have shown reduced purchases and consumption of unhealthy products and positive shifts in consumer behaviour [70, 77]. Given the regulations’ broad influence and interdependencies within the retail food system, a complex systems approach should be used to assess both process and outcomes [78–80]. Process aspects should include in-store compliance, shifts in supply chain relationships, changes in consumer-retailer interactions, and alterations in business-local authority dynamics. Additionally, it is essential to assess business adjustments, particularly the normalisation of reduced promotions on unhealthy foods, product substitutions, changes in food processing, and the introduction of new ingredients, as well as the effects on business profits. Finally, the intended effect of the regulations on purchasing and consumption patterns should be monitored, including whether there are shifts towards other unhealthy foods. Beyond these direct impacts, broader consequences such as evolving societal norms and demographic disparities must also be considered, particularly among lower-income individuals who rely on exempt smaller stores for their food purchases. Such collective evidence is necessary, particularly for complex policy interventions, as it is not possible to attribute long-term success to any one single policy intervention within a complex system [81].

### Strengths and limitations

A key strength of this research is that it was conducted during a period of significant advancement in obesity policy in the UK. The study findings provide an understanding of various factors influencing potential effectiveness of the regulations and highlight key aspects for monitoring and evaluation to aid future policy development and refinement. Both interviews and focus groups were utilised to collect data to provide flexibility to health experts and gain rich insights from focus groups where they interact with each other enriching the conversation.

By employing a purposive sampling approach, we successfully captured views from a diverse range of experts in the field. While the participating health experts included both academic and CSO representatives, we did not compare their perspectives. Future research could explore synergies and differences across stakeholder types to better understand how sectoral backgrounds influence views on the regulations and their potential to drive policy change.

It is widely recognised that a researcher’s background, perspectives, and experiences can influence data collection, analysis, and interpretation of qualitative data. To mitigate potential bias and enhance quality assurance, thematic analysis was conducted within an interdisciplinary team and the findings confirmed with a participant. The research reflects insights from a specific period of policy change. Health experts’ opinions on the effectiveness of policies might evolve after the regulations have been implemented and as new data emerges.

## Conclusions

This study highlights that, while the introduction of the Food (Promotion and Placement) regulations in the UK marks a significant policy shift towards recognising the role of businesses in contributing to obesity, several critical limitations must be addressed to ensure the regulations are effective. Key shortcomings include the reliance on the outdated 2004–2005 NPM, product and business exemptions, inadequate enforcement, and a lack of support for smaller stores. Improved funding mechanisms are necessary for long-term monitoring and for applying a systems-based approach to evaluating the regulations. Better integration and alignment of policies across various sectors of the food system is also needed to drive meaningful changes in retail marketing practices and to provide businesses with a consistent regulatory landscape.

## Supplementary Information

Additional file 1.

## Data Availability

The data collected for this study are not publicly available to protect the confidentiality of participants. Anonymised interview data can be made available upon reasonable request to the corresponding author pending approval.

## Abbreviations

UK: United Kingdom
HFSS: High fat, salt, or sugar
NPM: Nutrient profiling model
COREQ: Consolidated Criteria for Reporting Qualitative Studies
CSOs: Civil society organisations
NGOs: Non-governmental organisations
DHSC: Department of Health and Social Care
