# Supplementary material for: The Food (Promotion and Placement) regulations are beginning to shift the onus for healthier choices from individuals to businesses: in-depth perspectives from health experts
Source: BMC Med. 2025 Dec 17;23:686. doi: 10.1186/s12916-025-04484-2 (PMC12709698; doi:10.1186/s12916-025-04484-2)
Supplement: Supplementary file 1 — Additional file 1. [file 12916_2025_4484_MOESM1_ESM.docx]

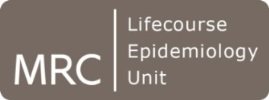

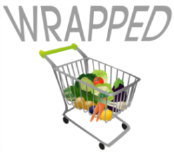


# MRC Lifecourse Epidemiology Unit

Southampton General Hospital

# Southampton S016 6YD

Telephone: 023 8120 4186


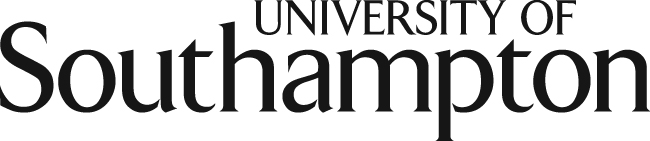


d d m m y y

**Health expert:**  **Date**: T**ime:**

**Focus group/Interview guide – Health experts**

**Introduction**

Hello, I’m a *[name]* from the University of Southampton. We are conducting research to understand your **views on the upcoming legislation** **restricting promotion and placement of unhealthy foods** in retail outlets.

For this research we will be asking some questions to understand your views as a health expert. We would like to record the meeting with your permission. The recording will be typed up and read by the research team and any names will be removed from the written version.

We encourage you to have your cameras on, but we understand if you would prefer to have them off. If you want to remain anonymous you can change your name at the bottom under participants. Mainly you will have your microphones on mute but do unmute when you would like to talk. You can also use the chat function if you would like to speak and raise your hand physically. It would be great if we could hear from all of you.

I will begin by asking you for your names for the purposes of the discussion only. Your names will not be reported at any point. So please be assured that your contribution today will remain anonymous and confidential in written reports. The discussion will last approximately 45 minutes. You can choose not to answer questions. If you wish to leave the discussion at any point, you are of course able to do that. My colleague “xx” will be observing and taking some notes.

We really value your contribution as your views will bring an important angle in terms of benefits and concerns related to this legislation.

**Prompting questions**

1. **What are your opinions of the legislation for restricting the promotion and placement of unhealthy foods (high fat, sugar and salt products) in retail store outlets?**

*Prompts*

- *Opinions on legislation for online retail*
- *Main benefits and/or concerns*

1. **How clear is the upcoming legislation?**
2. **How well do you think this legislation will be enforced?**
   1. **How might this differ across local authorities/regions**

*Prompts*

- *Effectiveness of fines in preventing non-compliance*
- *Workload issues for enforcers thus limiting effectiveness of enforcement*

1. **In your opinion, how could the enforcement of this legislation be supported?**
2. **What impact do you think the legislation will have on public health?**

*Prompts*

- *Unintended consequences for consumers, society or the government*
- *Public health impact changing overtime*

1. **What impact do you think the legislation will have on businesses?**
2. **What suggestions do you have to effectively measure the impact of this legislation?**
   1. **How might this differ across retailers, online/in-store etc?**

*Prompts*

- *Which data sources, duration of data, systems approach, qual/quant*
- *Supermarket layouts*
- *Consumer awareness*
- *Changes to societal norms*
- *Economic evaluation*

1. **How do you think consumers will respond to this legislation?**

**What differences in consumer response do you predict across demographic groups?**

*Prompts*

- *Different ethnic groups responses to the legislation*
- *Gender*
- *Socioeconomic groups*

1. **What improvements do you think could be made to this legislation to maximise the public health benefit, particularly** **to vulnerable groups?**

*Prompts*

- *Difference by business type and size*
- *Differences by in-store and online*
- *Concerns about alternative promotion strategies for promoting unhealthy foods*

1. **More generally, who do you think is responsible for supporting healthy eating?**

*Prompt*

- *Responsibility of retailers/food industry, government or individuals*

**End with:**

- Any last comments or thoughts that we haven’t yet discussed?
- Thank you for your time.
